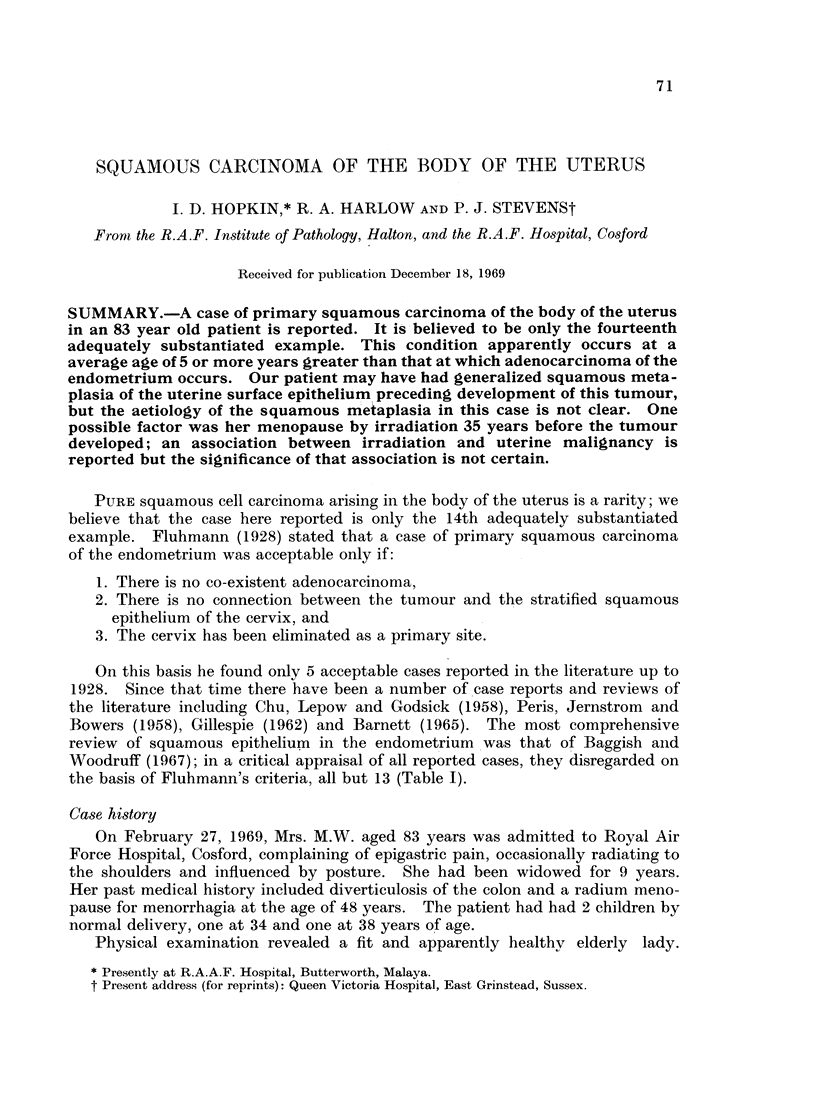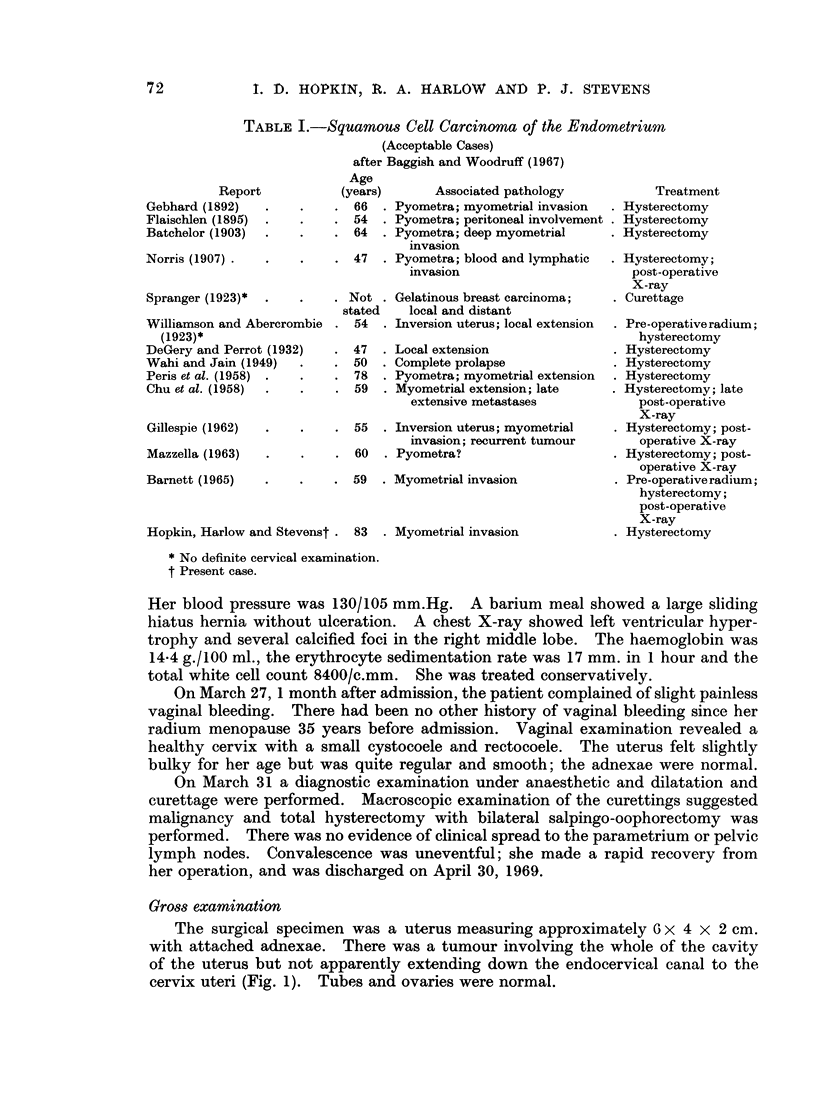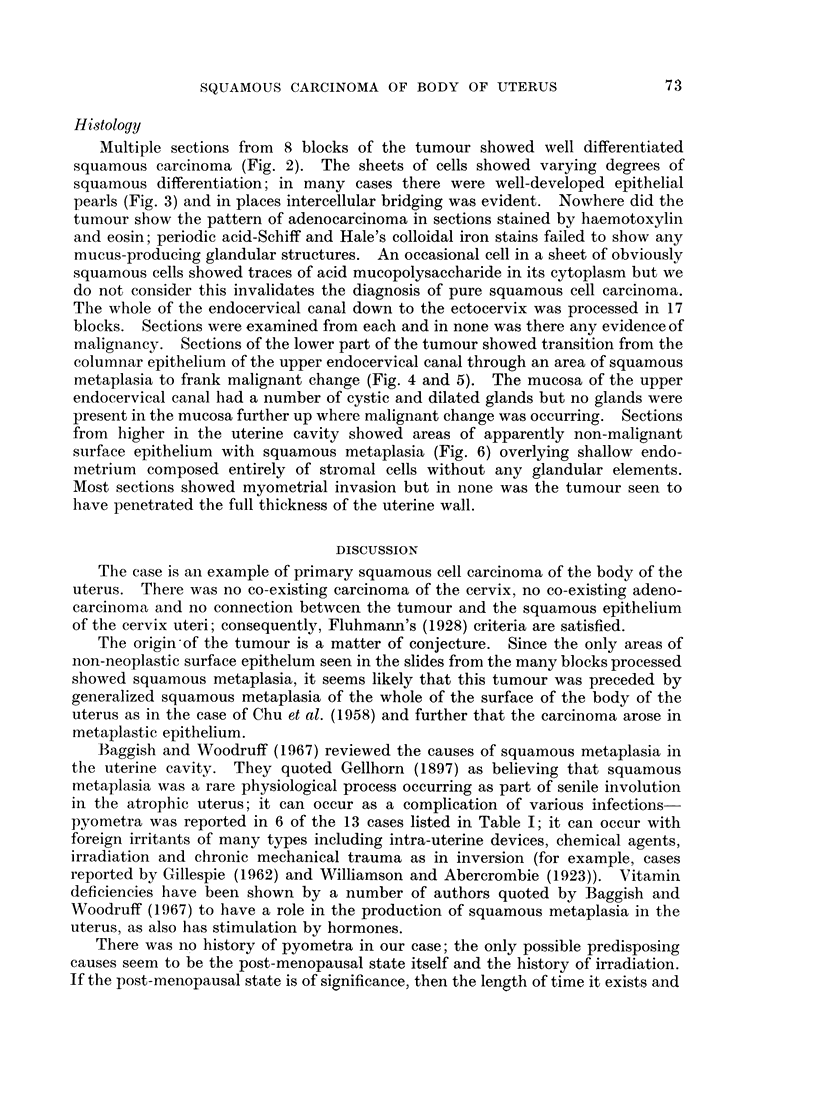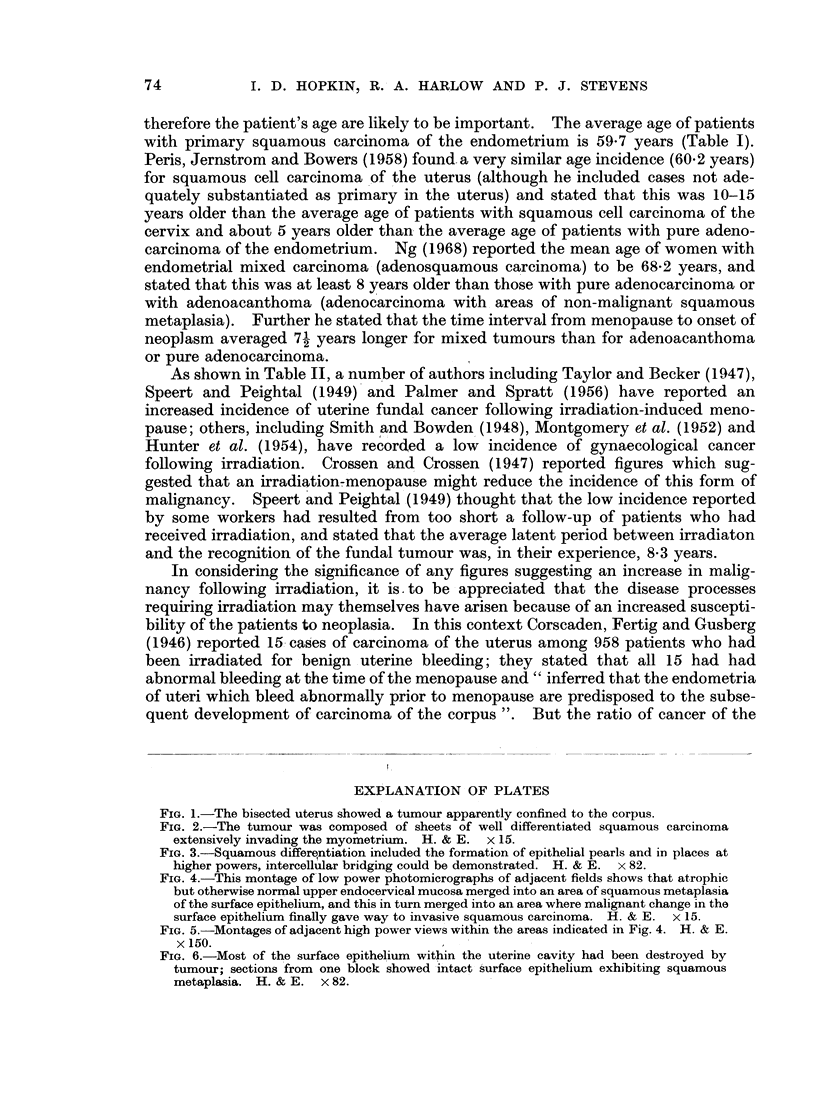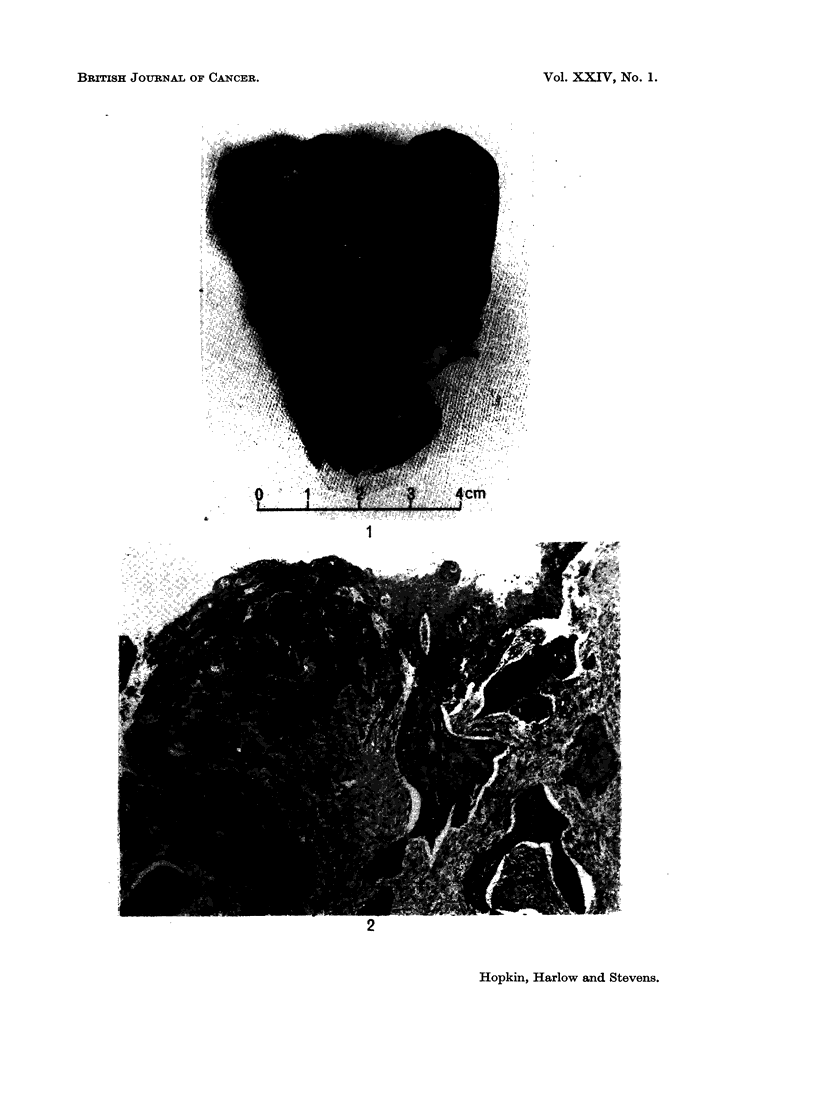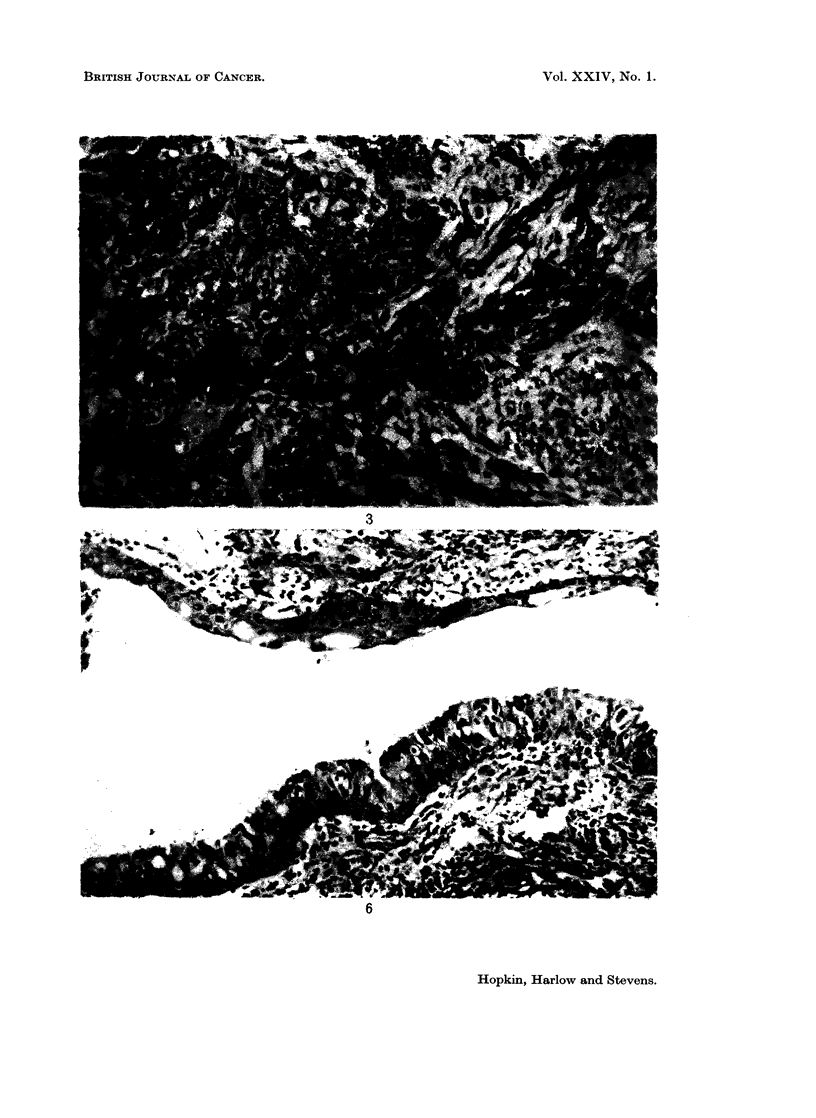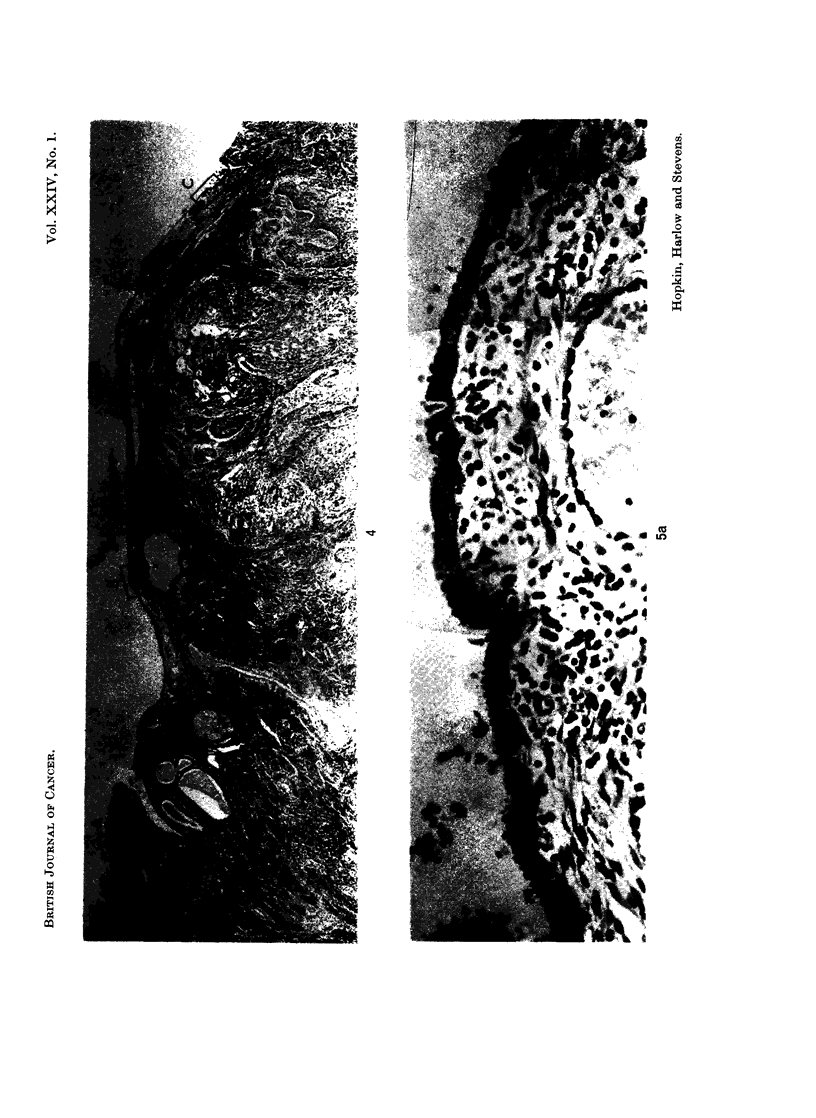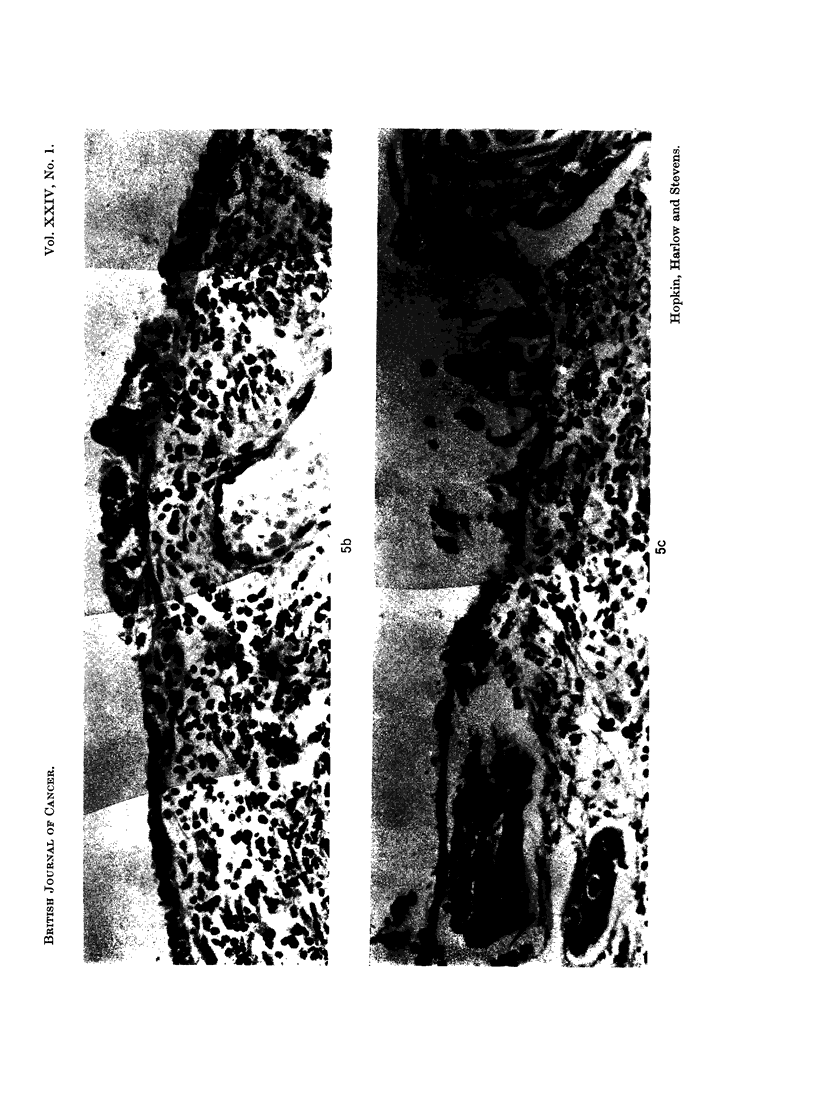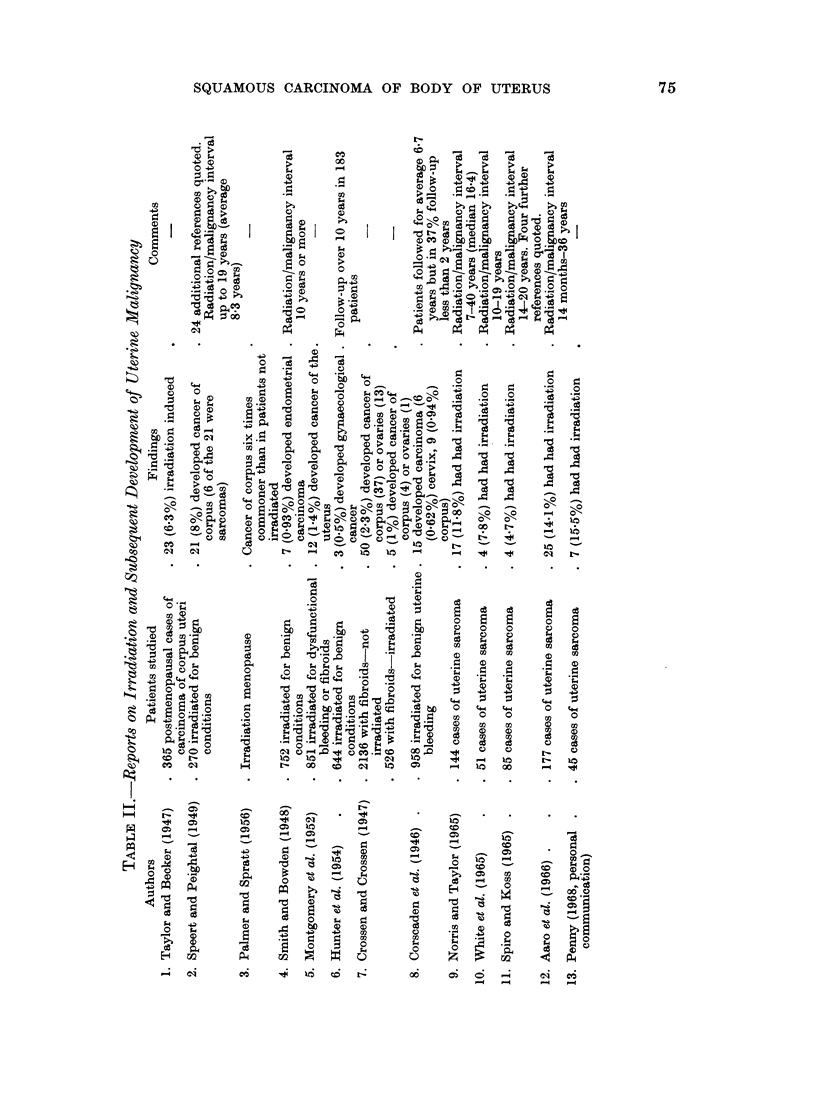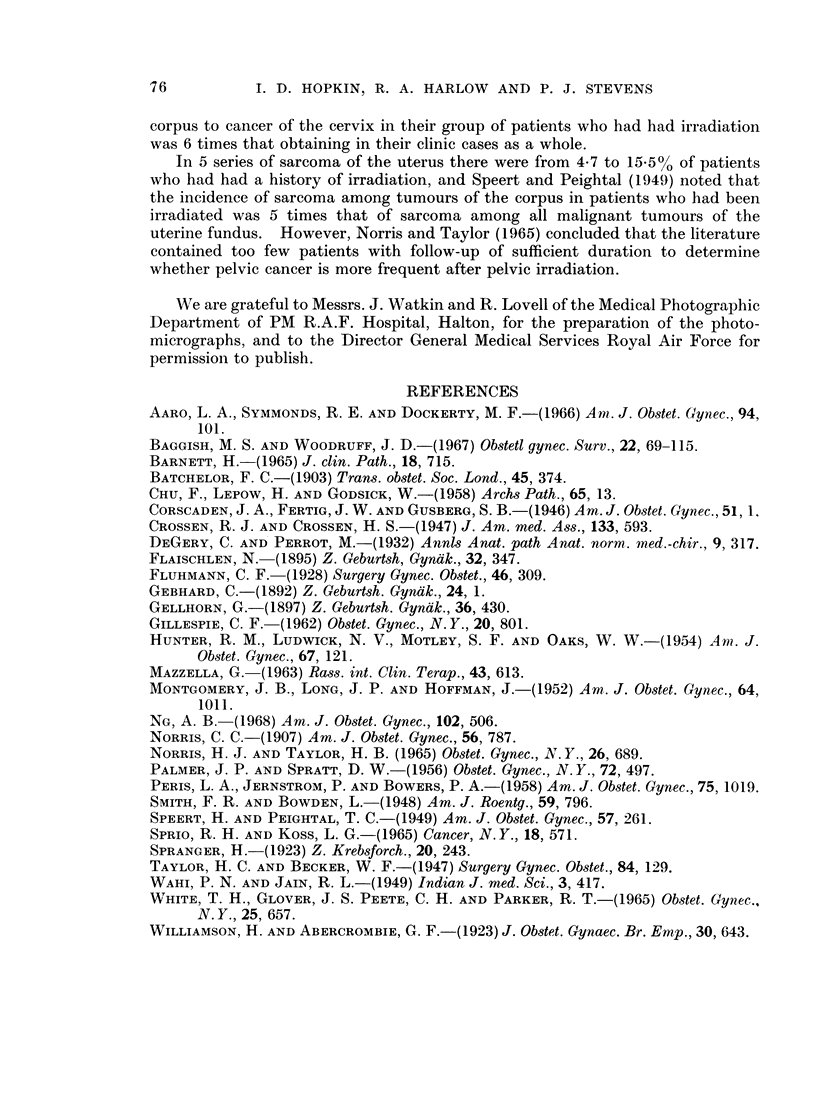# Squamous Carcinoma of the Body of the Uterus

**DOI:** 10.1038/bjc.1970.10

**Published:** 1970-03

**Authors:** I. D. Hopkin, R. A. Harlow, P. J. Stevens

## Abstract

**Images:**


### SQUAMOUS CARCINOMA OF THE BODY OF THE UTERUS

I. D. HOPKIN,* R. A. HARLOW AND P. J. STEVENS†

*From the R.A.F. Institute of Pathology, Halton, and the R.A.F. Hospital, Cosford*

Received for publication December 18, 1969

**SUMMARY.—A case of primary squamous carcinoma of the body of the uterus in an 83 year old patient is reported. It is believed to be only the fourteenth adequately substantiated example. This condition apparently occurs at a average age of 5 or more years greater than that at which adenocarcinoma of the endometrium occurs. Our patient may have had generalized squamous metaplasia of the uterine surface epithelium preceding development of this tumour, but the aetiology of the squamous metaplasia in this case is not clear. One possible factor was her menopause by irradiation 35 years before the tumour developed; an association between irradiation and uterine malignancy is reported but the significance of that association is not certain.**

PURE squamous cell carcinoma arising in the body of the uterus is a rarity; we believe that the case here reported is only the 14th adequately substantiated example. Fluhmann (1928) stated that a case of primary squamous carcinoma of the endometrium was acceptable only if:

1. There is no co-existent adenocarcinoma,
2. There is no connection between the tumour and the stratified squamous epithelium of the cervix, and
3. The cervix has been eliminated as a primary site.

On this basis he found only 5 acceptable cases reported in the literature up to 1928. Since that time there have been a number of case reports and reviews of the literature including Chu, Lepow and Godsick (1958), Peris, Jernstrom and Bowers (1958), Gillespie (1962) and Barnett (1965). The most comprehensive review of squamous epithelium in the endometrium was that of Baggish and Woodruff (1967); in a critical appraisal of all reported cases, they disregarded on the basis of Fluhmann's criteria, all but 13 (Table I).

*Case history*

On February 27, 1969, Mrs. M.W. aged 83 years was admitted to Royal Air Force Hospital, Cosford, complaining of epigastric pain, occasionally radiating to the shoulders and influenced by posture. She had been widowed for 9 years. Her past medical history included diverticulosis of the colon and a radium menopause for menorrhagia at the age of 48 years. The patient had had 2 children by normal delivery, one at 34 and one at 38 years of age.

Physical examination revealed a fit and apparently healthy elderly lady.

* Presently at R.A.A.F. Hospital, Butterworth, Malaya.
† Present address (for reprints): Queen Victoria Hospital, East Grinstead, Sussex.

TABLE I.—*Squamous Cell Carcinoma of the Endometrium*
(Acceptable Cases)
after Baggish and Woodruff (1967)

| Report | Age (years) | Associated pathology | Treatment |
|---|---|---|---|
| Gebhard (1892) | 66 | Pyometra; myometrial invasion | Hysterectomy |
| Flaischlen (1895) | 54 | Pyometra; peritoneal involvement | Hysterectomy |
| Batchelor (1903) | 64 | Pyometra; deep myometrial invasion | Hysterectomy |
| Norris (1907) | 47 | Pyometra; blood and lymphatic invasion | Hysterectomy; post-operative X-ray |
| Spranger (1923)* | Not stated | Gelatinous breast carcinoma; local and distant | Curettage |
| Williamson and Abercrombie (1923)* | 54 | Inversion uterus; local extension | Pre-operative radium; hysterectomy |
| DeGery and Perrot (1932) | 47 | Local extension | Hysterectomy |
| Wahi and Jain (1949) | 50 | Complete prolapse | Hysterectomy |
| Peris *et al.* (1958) | 78 | Pyometra; myometrial extension | Hysterectomy |
| Chu *et al.* (1958) | 59 | Myometrial extension; late extensive metastases | Hysterectomy; late post-operative X-ray |
| Gillespie (1962) | 55 | Inversion uterus; myometrial invasion; recurrent tumour | Hysterectomy; post-operative X-ray |
| Mazzella (1963) | 60 | Pyometra? | Hysterectomy; post-operative X-ray |
| Barnett (1965) | 59 | Myometrial invasion | Pre-operative radium; hysterectomy; post-operative X-ray |
| Hopkin, Harlow and Stevens† | 83 | Myometrial invasion | Hysterectomy |

* No definite cervical examination.

† Present case.

Her blood pressure was 130/105 mm.Hg. A barium meal showed a large sliding hiatus hernia without ulceration. A chest X-ray showed left ventricular hypertrophy and several calcified foci in the right middle lobe. The haemoglobin was 14·4 g./100 ml., the erythrocyte sedimentation rate was 17 mm. in 1 hour and the total white cell count 8400/c.mm. She was treated conservatively.

On March 27, 1 month after admission, the patient complained of slight painless vaginal bleeding. There had been no other history of vaginal bleeding since her radium menopause 35 years before admission. Vaginal examination revealed a healthy cervix with a small cystocoele and rectocoele. The uterus felt slightly bulky for her age but was quite regular and smooth; the adnexae were normal.

On March 31 a diagnostic examination under anaesthetic and dilatation and curettage were performed. Macroscopic examination of the curettings suggested malignancy and total hysterectomy with bilateral salpingo-oophorectomy was performed. There was no evidence of clinical spread to the parametrium or pelvic lymph nodes. Convalescence was uneventful; she made a rapid recovery from her operation, and was discharged on April 30, 1969.

*Gross examination*

The surgical specimen was a uterus measuring approximately 6 × 4 × 2 cm. with attached adnexae. There was a tumour involving the whole of the cavity of the uterus but not apparently extending down the endocervical canal to the cervix uteri (Fig. 1). Tubes and ovaries were normal.

*Histology*

Multiple sections from 8 blocks of the tumour showed well differentiated squamous carcinoma (Fig. 2). The sheets of cells showed varying degrees of squamous differentiation; in many cases there were well-developed epithelial pearls (Fig. 3) and in places intercellular bridging was evident. Nowhere did the tumour show the pattern of adenocarcinoma in sections stained by haemotoxylin and eosin; periodic acid-Schiff and Hale's colloidal iron stains failed to show any mucus-producing glandular structures. An occasional cell in a sheet of obviously squamous cells showed traces of acid mucopolysaccharide in its cytoplasm but we do not consider this invalidates the diagnosis of pure squamous cell carcinoma. The whole of the endocervical canal down to the ectocervix was processed in 17 blocks. Sections were examined from each and in none was there any evidence of malignancy. Sections of the lower part of the tumour showed transition from the columnar epithelium of the upper endocervical canal through an area of squamous metaplasia to frank malignant change (Fig. 4 and 5). The mucosa of the upper endocervical canal had a number of cystic and dilated glands but no glands were present in the mucosa further up where malignant change was occurring. Sections from higher in the uterine cavity showed areas of apparently non-malignant surface epithelium with squamous metaplasia (Fig. 6) overlying shallow endometrium composed entirely of stromal cells without any glandular elements. Most sections showed myometrial invasion but in none was the tumour seen to have penetrated the full thickness of the uterine wall.

### DISCUSSION

The case is an example of primary squamous cell carcinoma of the body of the uterus. There was no co-existing carcinoma of the cervix, no co-existing adenocarcinoma and no connection between the tumour and the squamous epithelium of the cervix uteri; consequently, Fluhmann's (1928) criteria are satisfied.

The origin of the tumour is a matter of conjecture. Since the only areas of non-neoplastic surface epithelum seen in the slides from the many blocks processed showed squamous metaplasia, it seems likely that this tumour was preceded by generalized squamous metaplasia of the whole of the surface of the body of the uterus as in the case of Chu *et al.* (1958) and further that the carcinoma arose in metaplastic epithelium.

Baggish and Woodruff (1967) reviewed the causes of squamous metaplasia in the uterine cavity. They quoted Gellhorn (1897) as believing that squamous metaplasia was a rare physiological process occurring as part of senile involution in the atrophic uterus; it can occur as a complication of various infections—pyometra was reported in 6 of the 13 cases listed in Table I; it can occur with foreign irritants of many types including intra-uterine devices, chemical agents, irradiation and chronic mechanical trauma as in inversion (for example, cases reported by Gillespie (1962) and Williamson and Abercrombie (1923)). Vitamin deficiencies have been shown by a number of authors quoted by Baggish and Woodruff (1967) to have a role in the production of squamous metaplasia in the uterus, as also has stimulation by hormones.

There was no history of pyometra in our case; the only possible predisposing causes seem to be the post-menopausal state itself and the history of irradiation. If the post-menopausal state is of significance, then the length of time it exists and therefore the patient's age are likely to be important. The average age of patients with primary squamous carcinoma of the endometrium is 59·7 years (Table I). Peris, Jernstrom and Bowers (1958) found a very similar age incidence (60·2 years) for squamous cell carcinoma of the uterus (although he included cases not adequately substantiated as primary in the uterus) and stated that this was 10–15 years older than the average age of patients with squamous cell carcinoma of the cervix and about 5 years older than the average age of patients with pure adenocarcinoma of the endometrium. Ng (1968) reported the mean age of women with endometrial mixed carcinoma (adenosquamous carcinoma) to be 68·2 years, and stated that this was at least 8 years older than those with pure adenocarcinoma or with adenoacanthoma (adenocarcinoma with areas of non-malignant squamous metaplasia). Further he stated that the time interval from menopause to onset of neoplasm averaged $7\frac{1}{2}$ years longer for mixed tumours than for adenoacanthoma or pure adenocarcinoma.

As shown in Table II, a number of authors including Taylor and Becker (1947), Speert and Peightal (1949) and Palmer and Spratt (1956) have reported an increased incidence of uterine fundal cancer following irradiation-induced menopause; others, including Smith and Bowden (1948), Montgomery *et al.* (1952) and Hunter *et al.* (1954), have recorded a low incidence of gynaecological cancer following irradiation. Crossen and Crossen (1947) reported figures which suggested that an irradiation-menopause might reduce the incidence of this form of malignancy. Speert and Peightal (1949) thought that the low incidence reported by some workers had resulted from too short a follow-up of patients who had received irradiation, and stated that the average latent period between irradiaton and the recognition of the fundal tumour was, in their experience, 8·3 years.

In considering the significance of any figures suggesting an increase in malignancy following irradiation, it is to be appreciated that the disease processes requiring irradiation may themselves have arisen because of an increased susceptibility of the patients to neoplasia. In this context Corscaden, Fertig and Gusberg (1946) reported 15 cases of carcinoma of the uterus among 958 patients who had been irradiated for benign uterine bleeding; they stated that all 15 had had abnormal bleeding at the time of the menopause and " inferred that the endometria of uteri which bleed abnormally prior to menopause are predisposed to the subsequent development of carcinoma of the corpus ". But the ratio of cancer of the

---

EXPLANATION OF PLATES

FIG. 1.—The bisected uterus showed a tumour apparently confined to the corpus.

FIG. 2.—The tumour was composed of sheets of well differentiated squamous carcinoma extensively invading the myometrium. H. & E. $\times 15$.

FIG. 3.—Squamous differentiation included the formation of epithelial pearls and in places at higher powers, intercellular bridging could be demonstrated. H. & E. $\times 82$.

FIG. 4.—This montage of low power photomicrographs of adjacent fields shows that atrophic but otherwise normal upper endocervical mucosa merged into an area of squamous metaplasia of the surface epithelium, and this in turn merged into an area where malignant change in the surface epithelium finally gave way to invasive squamous carcinoma. H. & E. $\times 15$.

FIG. 5.—Montages of adjacent high power views within the areas indicated in Fig. 4. H. & E. $\times 150$.

FIG. 6.—Most of the surface epithelium within the uterine cavity had been destroyed by tumour; sections from one block showed intact surface epithelium exhibiting squamous metaplasia. H. & E. $\times 82$.

1

2

3

6

4

5a

Hopkin, Harlow and Stevens.

5b

5c

Hopkin, Harlow and Stevens.

TABLE II.—*Reports on Irradiation and Subsequent Development of Uterine Malignancy*

| Authors | Patients studied | Findings | Comments |
|---|---|---|---|
| 1. Taylor and Becker (1947) | 365 postmenopausal cases of carcinoma of corpus uteri | 23 (6·3%) irradiation induced | — |
| 2. Speert and Peightal (1949) | 270 irradiated for benign conditions | 21 (8%) developed cancer of corpus (6 of the 21 were sarcomas) | 24 additional references quoted. Radiation/malignancy interval up to 19 years (average 8·3 years) |
| 3. Palmer and Spratt (1956) | Irradiation menopause | Cancer of corpus six times commoner than in patients not irradiated | — |
| 4. Smith and Bowden (1948) | 752 irradiated for benign conditions | 7 (0·93%) developed endometrial carcinoma | Radiation/malignancy interval 10 years or more |
| 5. Montgomery *et al.* (1952) | 851 irradiated for dysfunctional bleeding or fibroids | 12 (1·4%) developed cancer of the uterus | — |
| 6. Hunter *et al.* (1954) | 644 irradiated for benign conditions | 3 (0·5%) developed gynaecological cancer | Follow-up over 10 years in 183 patients |
| 7. Crossen and Crossen (1947) | 2136 with fibroids—not irradiated | 50 (2·3%) developed cancer of corpus (37) or ovaries (13) | — |
| | 526 with fibroids—irradiated | 5 (1%) developed cancer of corpus (4) or ovaries (1) | — |
| 8. Corscaden *et al.* (1946) | 958 irradiated for benign uterine bleeding | 15 developed carcinoma (6 (0·62%) cervix, 9 (0·94%) corpus) | Patients followed for average 6·7 years but in 37% follow-up less than 2 years |
| 9. Norris and Taylor (1965) | 144 cases of uterine sarcoma | 17 (11·8%) had had irradiation | Radiation/malignancy interval 7–40 years (median 16·4) |
| 10. White *et al.* (1965) | 51 cases of uterine sarcoma | 4 (7·8%) had had irradiation | Radiation/malignancy interval 10–19 years |
| 11. Spiro and Koss (1965) | 85 cases of uterine sarcoma | 4 (4·7%) had had irradiation | Radiation/malignancy interval 14–20 years. Four further references quoted. |
| 12. Aaro *et al.* (1966) | 177 cases of uterine sarcoma | 25 (14·1%) had had irradiation | Radiation/malignancy interval 14 months–36 years |
| 13. Penny (1968, personal communication) | 45 cases of uterine sarcoma | 7 (15·5%) had had irradiation | — |

corpus to cancer of the cervix in their group of patients who had had irradiation was 6 times that obtaining in their clinic cases as a whole.

In 5 series of sarcoma of the uterus there were from 4·7 to 15·5% of patients who had had a history of irradiation, and Speert and Peightal (1949) noted that the incidence of sarcoma among tumours of the corpus in patients who had been irradiated was 5 times that of sarcoma among all malignant tumours of the uterine fundus. However, Norris and Taylor (1965) concluded that the literature contained too few patients with follow-up of sufficient duration to determine whether pelvic cancer is more frequent after pelvic irradiation.

We are grateful to Messrs. J. Watkin and R. Lovell of the Medical Photographic Department of PM R.A.F. Hospital, Halton, for the preparation of the photomicrographs, and to the Director General Medical Services Royal Air Force for permission to publish.

### REFERENCES

Aaro, L. A., Symmonds, R. E. and Dockerty, M. F.—(1966) *Am. J. Obstet. Gynec.*, **94**, 101.
Baggish, M. S. and Woodruff, J. D.—(1967) *Obstetl gynec. Surv.*, **22**, 69–115.
Barnett, H.—(1965) *J. clin. Path.*, **18**, 715.
Batchelor, F. C.—(1903) *Trans. obstet. Soc. Lond.*, **45**, 374.
Chu, F., Lepow, H. and Godsick, W.—(1958) *Archs Path.*, **65**, 13.
Corscaden, J. A., Fertig, J. W. and Gusberg, S. B.—(1946) *Am. J. Obstet. Gynec.*, **51**, 1.
Crossen, R. J. and Crossen, H. S.—(1947) *J. Am. med. Ass.*, **133**, 593.
DeGery, C. and Perrot, M.—(1932) *Annls Anat. path Anat. norm. med.-chir.*, **9**, 317.
Flaischlen, N.—(1895) *Z. Geburtsh, Gynäk.*, **32**, 347.
Fluhmann, C. F.—(1928) *Surgery Gynec. Obstet.*, **46**, 309.
Gebhard, C.—(1892) *Z. Geburtsh. Gynäk.*, **24**, 1.
Gellhorn, G.—(1897) *Z. Geburtsh. Gynäk.*, **36**, 430.
Gillespie, C. F.—(1962) *Obstet. Gynec., N.Y.*, **20**, 801.
Hunter, R. M., Ludwick, N. V., Motley, S. F. and Oaks, W. W.—(1954) *Am. J. Obstet. Gynec.*, **67**, 121.
Mazzella, G.—(1963) *Rass. int. Clin. Terap.*, **43**, 613.
Montgomery, J. B., Long, J. P. and Hoffman, J.—(1952) *Am. J. Obstet. Gynec.*, **64**, 1011.
Ng, A. B.—(1968) *Am. J. Obstet. Gynec.*, **102**, 506.
Norris, C. C.—(1907) *Am. J. Obstet. Gynec.*, **56**, 787.
Norris, H. J. and Taylor, H. B. (1965) *Obstet. Gynec., N.Y.*, **26**, 689.
Palmer, J. P. and Spratt, D. W.—(1956) *Obstet. Gynec., N.Y.*, **72**, 497.
Peris, L. A., Jernstrom, P. and Bowers, P. A.—(1958) *Am. J. Obstet. Gynec.*, **75**, 1019.
Smith, F. R. and Bowden, L.—(1948) *Am. J. Roentg.*, **59**, 796.
Speert, H. and Peightal, T. C.—(1949) *Am. J. Obstet. Gynec.*, **57**, 261.
Sprio, R. H. and Koss, L. G.—(1965) *Cancer, N.Y.*, **18**, 571.
Spranger, H.—(1923) *Z. Krebsforch.*, **20**, 243.
Taylor, H. C. and Becker, W. F.—(1947) *Surgery Gynec. Obstet.*, **84**, 129.
Wahi, P. N. and Jain, R. L.—(1949) *Indian J. med. Sci.*, **3**, 417.
White, T. H., Glover, J. S. Peete, C. H. and Parker, R. T.—(1965) *Obstet. Gynec., N.Y.*, **25**, 657.
Williamson, H. and Abercrombie, G. F.—(1923) *J. Obstet. Gynaec. Br. Emp.*, **30**, 643.